# Association Between Neonatal Urinary Tract Infection and Risk of Childhood Allergic Rhinitis: Erratum

**DOI:** 10.1097/01.md.0000499791.86340.b0

**Published:** 2015-10-30

In the article “Association Between Neonatal Urinary Tract Infection and Risk of Childhood Allergic Rhinitis”,^1^ which appeared in Volume 94, Issue 38 of *Medicine*, Dr. I-Ching Lin's name was spelled incorrectly in the by line. The article has since been corrected online.
